# Prolongation of Tick-Borne Encephalitis Cycles in Warmer Climatic Conditions

**DOI:** 10.3390/ijerph16224532

**Published:** 2019-11-15

**Authors:** Petr Zeman

**Affiliations:** Medical Laboratories, Konevova 205, 130 00 Prague, Czech Republic; zeman3@post.cz

**Keywords:** tick-borne encephalitis, disease dynamics, time-series analysis, climate change

## Abstract

Tick-borne encephalitis exhibits profound inter-annual fluctuations in incidence. Previous studies showed that three-fifths of the variation can be explained in terms of four superimposed oscillations: a quasi-biennial, triennial, pentennial, and a decadal cycle. This study was conducted to determine how these cycles could be influenced by climate change. Epidemiological data, spanning from the 1970s to the present, and originating from six regions/countries bridging Scandinavia and the Mediterranean, represented a temporal/latitudinal gradient. Spectral analysis of time series was used to determine variation in the cycles’ length/amplitude with respect to these gradients. The analysis showed that—whereas the lengths of the shorter cycles do not vary substantially—cycles in the decadal band tend to be longer southwards. When comparing the disease’s oscillations before- and after the mid-1990s, a shift towards longer oscillations was detected in the pentennial–decadal band, but not in the biennial– triennial band. Simultaneously, oscillations in the latter band increased in intensity whereas the decadal oscillations weakened. In summary, the rhythm of the cycles has been altered by climate change. Lengthened cycles may be explained by prolonged survival of some animal hosts, and consequently greater inertia in herd immunity changes, slowing down a feedback loop between the herd immunity and amount of virus circulating in nature.

## 1. Introduction

Tick-borne encephalitis (TBE) is the most serious vector-borne disease in Europe (EU). About 2500 cases are reported in EU annually, 95% of them require hospitalization, 40–50% sustain long-term sequelae, and approximately every 100th patient dies [1,2]. Its causative agent is the tick-borne encephalitis virus (*Flaviviridae, Flavivirus*) (TBEV) belonging (in most parts of EU) to the (West) European subtype of the virus (TBEV-Eu) transmitted by the tick *Ixodes ricinus* L., also regarded as the virus’s main reservoir [3]. Small murid rodents are the principal maintenance- (or bridge-) hosts; the role of other animals, particularly ruminants, in TBEV circulation is under dispute, they, at least, regulate the *I. ricinus* population and contribute to transmission through consumption of untreated milk by the alimentary route [4]. There is no specific antiviral treatment available, and the main protection is vaccination and risk avoidance [5,6].

Over its entire distributional range, TBE exhibits unstable dynamics and profound inter-annual fluctuations in disease case numbers, which complicates prognosis for preventive purposes. Most of the proposed predictive models of TBE transmission risk rely on lagged correlation between either TBE incidence itself or the vector’s abundance and some indicative climatic variables (e.g., mean annual temperature, precipitation) or population estimates of key hosts (e.g., murid rodents, their predators as a proxy), or both, observed 1–2 years before. Validity of such a prediction is, of course, contingent on available covariate data and limited to 1–1.5 years ahead [7,8,9].

Previous studies showed that three-fifths of the variation in TBE incidence can be explained in terms of four superimposed oscillations: A quasi-biennial, triennial, pentennial, and a decadal cycle. The cycles are highly synchronous over large geographic areas and can be interpreted as self-oscillations of components of the disease system (e.g., cyclic countercheck of herd immunity and amount of circulating virus, population cycles of hosts and ticks) that are periodically synchronized/modulated by external factors (e.g., climate swings, mast events). Some long-term forecast (≤4 yrs.) is thus possible by assessing these oscillations from TBE incidence data, and by their projection onwards [10,11].

Similar interannual cyclicity of some zoonotic disease outbreaks have been demonstrated to be altered by climate change—conceivably through the climate’s strong influence upon ecosystems and the natural background of these diseases [12,13,14]. Nothing is known as yet about effects of climate change upon the periodic patterns in TBE morbidity even though it is an important aspect in the disease’s forecasting. A certain impact can be, nevertheless, expected in view of the affection of the population dynamics of some important hosts involved in the disease’s circulation [15]. This study was conducted to determine how the TBE cycles could be influenced by the present-day climate change.

## 2. Materials and Methods

### 2.1. Epidemiological Data

TBE incidence series from Sweden, East Germany, the Czech Republic, Austria, Slovenia, and North-East Italy were selected for the purpose of this study. They constitute a temporal gradient between the early 1970s and the present, and a latitudinal gradient between Scandinavia and the Mediterranean—along the 13th meridian east across a zone where TBE is exclusively caused by TBEV-Eu and transmitted by *I. ricinus*. The data originated from national surveillance systems, the details of which are summarized in Table 1.

Data on the background populations were obtained from the national offices of statistics. Vaccination against TBEV was disregarded in all countries/regions except for Austria where the vaccination coverage is high and well documented, and where only the unvaccinated fraction of the population in each particular year was considered for calculating the incidence.

### 2.2. Statistical Analysis

The study hypothesis is that—apart from random variation—there exist systematic shifts in the TBE cycles’ length and/or amplitude along the latitudinal gradient, and as the climate warming progresses, that could be discerned in long-term means. Prior to analysis, the epidemiological data were ‘stationarized’, i.e., modified to eliminate any trend (i.e., a long-term change in the mean incidence) and to stabilize variance, utilizing the empirical mode decomposition (EMD) method [21]. Unmodified data was used whenever the actual level of incidence was important.

Continuous wavelet transformation (CW), utilizing the Morlet wavelet, was applied to convert the one-dimensional incidence series into their two-dimensional time-frequency representations [22]. CW gave output data in the form of time-frequency arrays of quantities (e.g., power and phase) characterizing how the spectrum of oscillations varied over the period of observation. The obtained sequential transitory power spectra were summed along the time dimension to produce an average power spectrum for the whole period. Eventually, the average power spectra of the six regions were collated to show up any effect of latitude. To evaluate changes in the time axis direction, the period of observation (whenever sufficiently long) was split into two equally long segments before and after the mid-1990s (thereafter referred to as ‘pre-‘ and ‘post-warming’ periods) for which the average power spectra were calculated separately and compared to each other. A nonparametric bootstrap was used to test the null hypothesis (H0) that the pre- and post-warming spectra have the same distribution of frequencies. A layout of the analysis is provided as Supplementary Materials.

All computations were done in the R 3.4.3 environment utilizing the EMD 1.5.7 [23] and WaveletComp 1.1 [24] extension packages.

## 3. Results

### 3.1. Effects of Latitude

**Figure 1:**
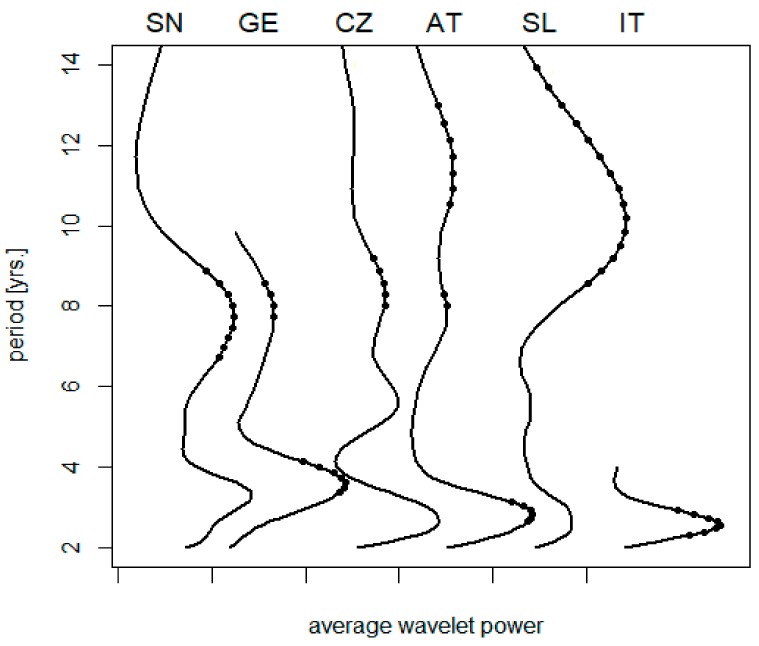
A collation of average wavelet power spectra of TBE fluctuations: (SN) Sweden, (GE) E. Germany, (CZ) the Czech Rep., (AT) Austria, (SL) Slovenia, and (IT) NE Italy; dots indicate where the signal exceeds random noise with 95% probability (truncated spectra are due to data shortage). For presentation purposes, the diagrams were individually scaled to have the same height of maxima, origins of the scales are marked on the horizontal axis. Note that—unlike the biennial–triennial oscillations—the cycle lengths in the decadal band exhibit a tendency to prolong from the north to south.

A climatic gradient along the 13th meridian is illustrated in Table 2. Corresponding variations in the spectrum of TBE oscillations are summarized in Figure 1. Short-term cycles collectively (biennial–triennial band) exhibit a relatively stable length and no clear-cut pattern relatable to latitude, although the northernmost regions show somewhat longer oscillations (but the series is either short (E. Germany) or the result statistically insignificant (Sweden)). The pentennial cycle is only slightly developed or even non-detectable in some regions; where discernible, it is suggestive of some prolongation southwardly. Eventually, the oscillations in the decadal band exhibit a distinct trend towards longer periods in warmer regions, ranging between <8 and >11 yrs. in the north and south, respectively (Spearman’s rho = 0.9, *p* = 0.017).

### 3.2. Longitudinal Shifts

**Figure 2:**
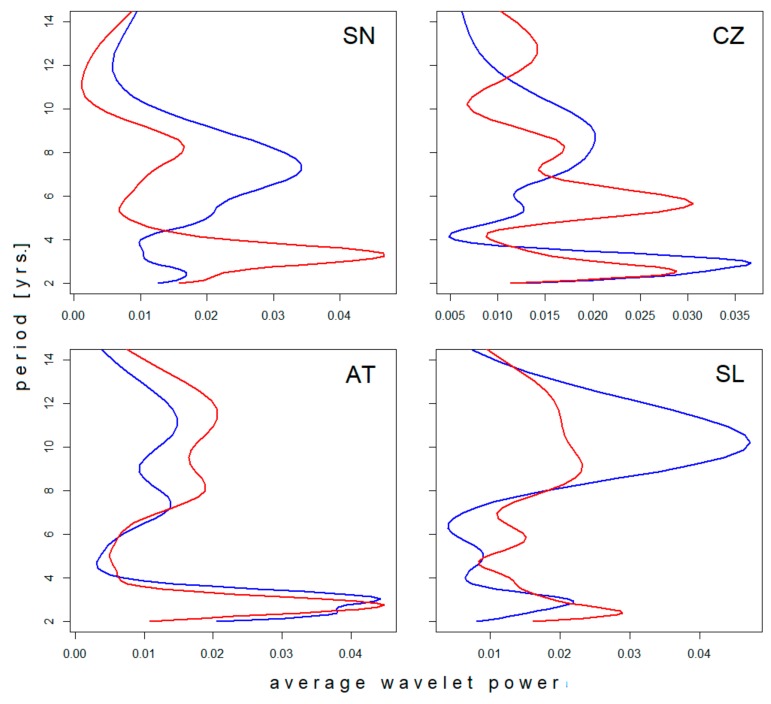
Comparison of average wavelet power spectra of tick-borne encephalitis (TBE) fluctuations in (blue) the pre-warming- and (red) post-warming periods in (SN) Sweden, (CZ) the Czech Rep., (AT) Austria, and (SL) Slovenia. For presentation, the spectra were normalized resulting in unitary area under the curve. Note a systematic shift towards longer periods of oscillation in the pentennial–decadal band contrasting with only erratic variations in the biennial–triennial band (best seen in Austria).

Four of the six regional incidence series were long enough to evince changes in the time axis direction. Differences between the spectra of oscillations in the ‘pre-’ and ‘post-warming’ periods are documented in Figure 2. In agreement with the neutral effect of latitude, oscillations in the biennial–triennial band do not exhibit any systematic shift with climate’s warming but rather a slight erratic variation across all climatic zones. Similarly, where detectable (the Czech Republic and Slovenia), the pentennial cycle suggests a prolongation in time. Within the decadal band, multiple modes of oscillation are manifested as more or less pronounced dichotomy/bimodality in the spectrum, apparently without any link to latitude or period. Where the pattern was stable—regardless of whether unimodal or bimodal (Sweden and Austria, respectively)—a shift towards longer oscillations is evident. Where dichotomy developed in the course of observation (the Czech Republic and Slovenia), the mean period length of the successor oscillations turns out to be somewhat longer than the forerunning period length (although the difference is small and scarcely demonstrable in Slovenia).

**Figure 3:**
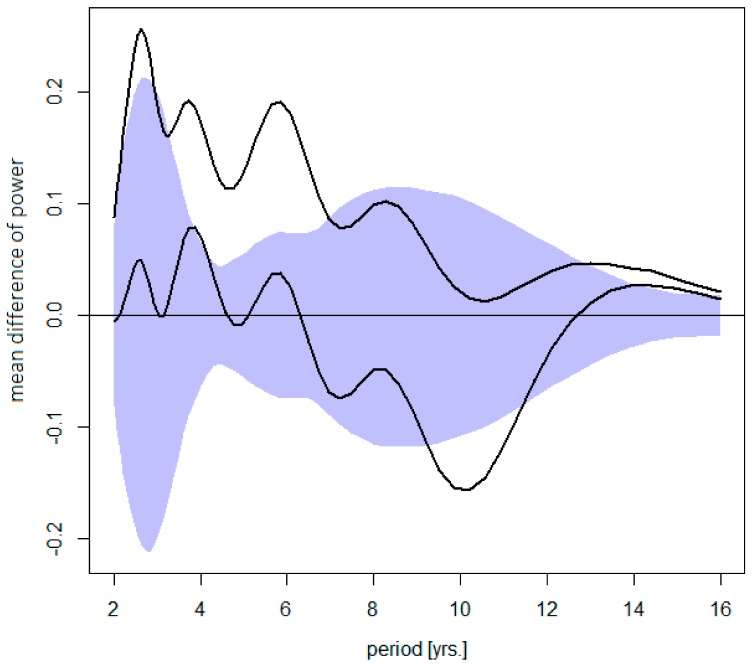
Difference between the post- and pre-warming average wavelet power spectra of TBE fluctuations (the former minus the latter)—shown is the mean difference for Sweden, the Czech Rep., Austria, and Slovenia altogether (upper diagram) counting in the trend, or (lower diagram) for stationarized data, plotted together with a 95% bootstrap confidence band for the mean difference under H0 (R = 1000 simulations). The dominating quasi-biennial-, triennial-, pentennial-, and the oscillations in the decadal-band are discernible. Note that (upper) an increment in power gradually decreases from the shortest to the longest oscillations (i.e., short cycles contribute the most to an overall incidence rise ), and that (lower) a positive- next to a negative-signed deviations in the decadal band testifies for a shift towards longer periods.

Changes in the TBE cycles with respect to their amplitude and contribution to an overall disease variance are summarized in Figure 3. It is evident that, in absolute terms, an increment of power between the ‘pre-’ and ‘post-warming’ periods systematically decreases from the shortest to the longest oscillations. In relative terms, the short cycles became, on average, augmented, and the long cycles damped, compared to the ‘pre-warming’ period.

### 3.3. Local Effects

**Figure 4:**
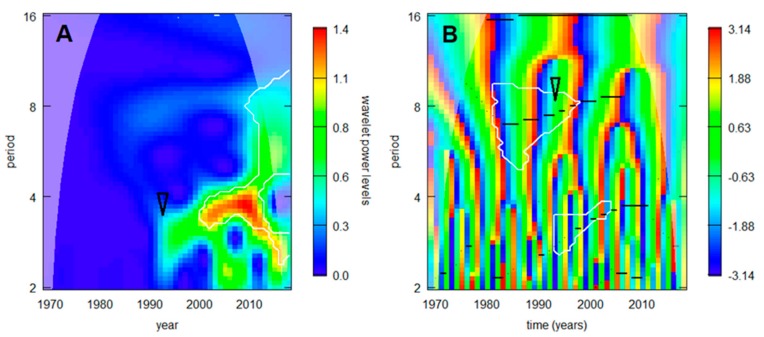
TBE dynamics in the northernmost situated Sweden in terms of (**A**) wavelet power calculated for the incidence series inclusive of trend, and (**B**) image of phases calculated for the stationarized data. White lines delimit domains where the signal exceeds random noise with 95% probability, the darker, ‘cones of influence’ indicate where the analysis is free of edge effects, and black lines in B show ‘ridges’—i.e., trace instantaneous frequencies of the dominating cycles (from the bottom upwards can be seen—quasi-biennial, triennial, and octennial/decadal oscillations). Noticeable is a turn in the 1990s accompanied with sudden increase in power (and lengthening) of the triennial cycle and accelerated prolongation of the decadal cycle (arrows).

Out of all regional TBE incidence series (showing relatively smooth development of the cycles’ spectra), the most abrupt change was detected in the northernmost series from Sweden (Figure 4). A noticeable turn occurred during the 1990s, characterized—in the main—by a sudden enhancement of the triennial cycle (visible also in Figure 2) and an accelerated prolongation of the decadal cycle.

## 4. Discussion

The analysis revealed consistent changes in the pattern of TBE oscillations attributable to temperature increase both in the latitudinal and temporal directions. Although other factors, such as precipitation, might have a certain effect as well, they were neglected in this study as no clear-cut gradient is recognizable across the study area and the period of observation. Various human activities also play an important role in TBE variation (e.g., [26]), however, their effects are, in principle, aperiodic, are unlikely to underlie a continuous geographical gradient, and had largely been removed by preprocessing the data. The anomaly detected in the Swedish data notably corresponds with a period of particularly rapid temperature increase in Scandinavia between 1995 and 2003 [27]. From the various factors considered, climate warming thus appears as the most probable driving factor of the changes in TBE oscillations.

Of the two alternative mechanisms explaining the rather coherent periodic patterns in the TBE dynamics over large areas—i.e., large-scale climatic forcing under teleconnection influence vs local-scale auto-oscillations synchronised externally—the latter conforms better with the epidemiological data. This can be documented by the inability of the North Atlantic Oscillation (NAO)—a prominent teleconnection relevant to the course of weather over Europe—to explain variations in TBE incidence in the Czech Republic and Sweden [8,28]. By contrast, self-oscillations can account for three-fifths of the variance in the disease fluctuations in Austria, Bavaria, the Czech Republic, and Switzerland [11]. Diverging auto-oscillations and intermittent synchronising events, e.g., mast years, adequately explain the alternation of phases of marked synchrony and asynchrony discerned in the TBE incidence series of central Europe [10]. Autonomous oscillations rather than a coherent teleconnection pattern also better tally with the discordance of the pentennial–decadal cycles at different latitudes revealed in this analysis. Eventually, the appearance of cycle splits within the decadal band in the course of observation further supports the self-oscillation hypothesis.

In physics, frequency shifts are most simply explained by allowing for a ‘friction factor’ in an oscillating system. By slowing down or speeding up the processes within the system (if friction is increased or reduced, respectively), an actual period of oscillation shifts somewhat off the system’s typical frequency. This concept can explain the shifting periodicity in TBE, as well. However, can the TBE disease system indeed establish autonomous oscillations? Preconditions are met immediately for multiple components of the system: Host populations are generally subject to density-dependent/predator-prey cycling, and a feedback also exists between population density of ticks and their resources due to acquired host resistance to tick feeding (e.g., [29]). Theoretical studies showed that tick populations would spontaneously oscillate even under constant conditions [30,31]. Another condition prone to oscillations is a reciprocal feedback between immunity of hosts and amount of circulating virus. Longitudinal surveys of TBEV prevalence in ticks, and of antibody prevalence in rodents, showed variations with a factor of 2–3 on a multiannual scale, which is in line with the assumed oscillation [18,32]. In endemic areas, high prevalence of TBEV-antibodies in roe deer (e.g., [33])—a key bloodmeal source for *I. ricinus*—is also suggestive of a regulatory effect upon TBEV circulation, though the hitherto data are too short to support true oscillations.

The diverse feedback loops and complexity of the disease system are reflected in the structured spectrum of TBE cycles and their differential response to climate warming. While the shorter, biennial–triennial cycles—associated most likely with population dynamics of species with a short-lifespan, i.e., ticks, rodents, and with their interactions—showed unchanged length and a moderate intensification, the longer cycles in the pentennial–decadal band—associable with long-lived hosts, e.g., deer—varied directly in the cycle length, and indirectly in power, with the temperature increase. One explanation of the differences is, of course, a methodical artefact as the resolution of this analysis is limited and subtle changes in the biennial–triennial band can have been overlooked. Alternatively, differences in the species’ biology, how they are affected by climate change, and how they cope with it could be responsible. For example, *I. ricinus* is ‘niche tracking’, i.e., it slowly moves with the shifted climatic envelope to maintain its ecological niche—in this fashion, it moved to higher altitudes in the Mediterranean [34], or further to the north at the opposite end of the latitudinal gradient in Scandinavia (e.g., [35]). It thus occupies environmental conditions that are largely static regardless of climate change, which may be a clue as to why the presumably ‘tick/rodent’ short cycles are relatively invariable.

In turn, roe deer—as a typical representative of large hosts—is ‘niche switching’, i.e., it adaptively changes environment in the course of a year to avoid harsh climatic conditions and take advantage of improved forage [36]. It can benefit from climate warming through reduced mortality, increased individual fitness, and population growth [37]. This could explain why the presumably ‘deer’ long cycles are more influenced by climate change. However, why the oscillations in the pentennial–decadal band are damped rather than boosted and what represents the ‘friction factor’? A possible explanation may lie in the feedback loop between herd immunity and amount of circulating virus, and in the demographic impact of climate change upon host populations. For example, the increasingly early onset of spring but inflexible, photoperiod-dependent breeding strategy in roe deer are responsible for juvenile mortality due to a delay of parturition and lactation behind the peak of plant productivity, whereby the population growth is to a greater extent driven by prolonged survival in adulthood [38]. Slowed down recruitment and increased longevity lead to greater inertia in herd immunity changes, and can explain prolongation of associated disease cycles. Geographical variability of this factor and regional differences in host synusiae can readily account for the existence of multiple modes of oscillation within the decadal band.

## 5. Conclusions

The present study showed that not only the spatial distribution but also the rhythm of TBE fluctuations has been altered by climate change. This is congruent with observations of similar changes in other zoonotic diseases throughout the world. The effect of temperature upon TBE cycles is relevant to disease forecasting and should be taken into consideration in predictive models. The tendency of worse predictable short cycles to increase in intensity and simultaneously a fade of better predictable long cycles may, however, impair accuracy of long-term forecasts in the future.

Self-oscillations of the disease system rather than an atmospheric teleconnection influence (‘atmospheric bridge’) comply better with the observed patterns in the epidemiologic data. This fact challenges the common notion that the processes in the disease system are (just) a play of external forces. More data from longitudinal epidemiologic and ecologic studies will be needed to elucidate the mechanisms underlying the TBE cycles.

## Figures and Tables

**Table 1:** Epidemiological data.

Region	Centroid Northing	Centroid Easting	Period	Cases	Authority	References
Sweden	59.5	15.0	1969–2018	5369	Public Health Agency of Sweden (Folkhälsomyndigheten)	[16,17]
E. Germany ^1^	52.5	13.4	1993–2017	201	Robert Koch Institute	[18]
Czech Rep.	49.8	15.5	1971–2018	22,716	National Institute of Public Health (SZU)	
Austria	47.7	14.9	1970–2018	9746	Zentrum für Virologie, Med. Universität Wien	[19]
Slovenia	46.2	14.9	1972–2018	8710	National Institute of Public Health (NIJZ)	[16]
NE Italy ^2^	46.2	12.2	2000–2013	367	n.a.	[20]

^1^ Mecklenburg-W Pomerania, Brandenburg, Berlin, Saxony, Saxony-Anhalt.; ^2^ Friuli Venezia Giulia, Trentino Alto Adige, Veneto.

**Table 2:** The latitudinal gradient in terms of average temperature and the number of freezing days at selected sites in the periods indicated [25]. Note irregularities related to altitude and continentality.

Region/Country	Site	Temperature			No. Freezing Days/y		
		1900–1999	2000–2018	Increase	1900–1999	2000–2018	Decrease
Sweden	Uppsala	5.9	7	1.1	77.5	60.6	16.9
	Stockholm	6.4	7.5	1.1	63.3	49.4	13.9
E Germany	Berlin	9.2	10.2	1	35.6	27.2	8.4
	Dresden	7.9	9.1	1.2	48.2	37.2	11
Czech Rep.	Prague	8	9.1	1.1	49.1	41.1	8
	Brno	8.6	9.6	1	46.2	40.3	5.9
Austria	Vienna	9	10.1	1.1	43.1	35.3	7.8
	Graz	8.1	8.9	0.8	53.4	46.3	7.1
Slovenia	Maribor	9.5	10.4	0.9	40.1	34.6	5.5
	Ljubljana	8.8	9.9	1.1	46.9	36.8	10.1
NE Italy	Belluno	7.8	8.9	1.1	51.3	37.1	14.2
	Trieste	11.4	12.6	1.2	20.9	9.8	11.1

## Supplementary Materials

The following is available online at https://www.mdpi.com/1660-4601/16/22/4532/s1: File S1: The analysis workflow.
