# Supplementary material for: Prolongation of Tick-Borne Encephalitis Cycles in Warmer Climatic Conditions
Source: Int J Environ Res Public Health. 2019 Nov 15;16(22):4532. doi: 10.3390/ijerph16224532 (PMC6888212; doi:10.3390/ijerph16224532)
Supplement: Supplementary file 1 [file ijerph-16-04532-s001.pdf]

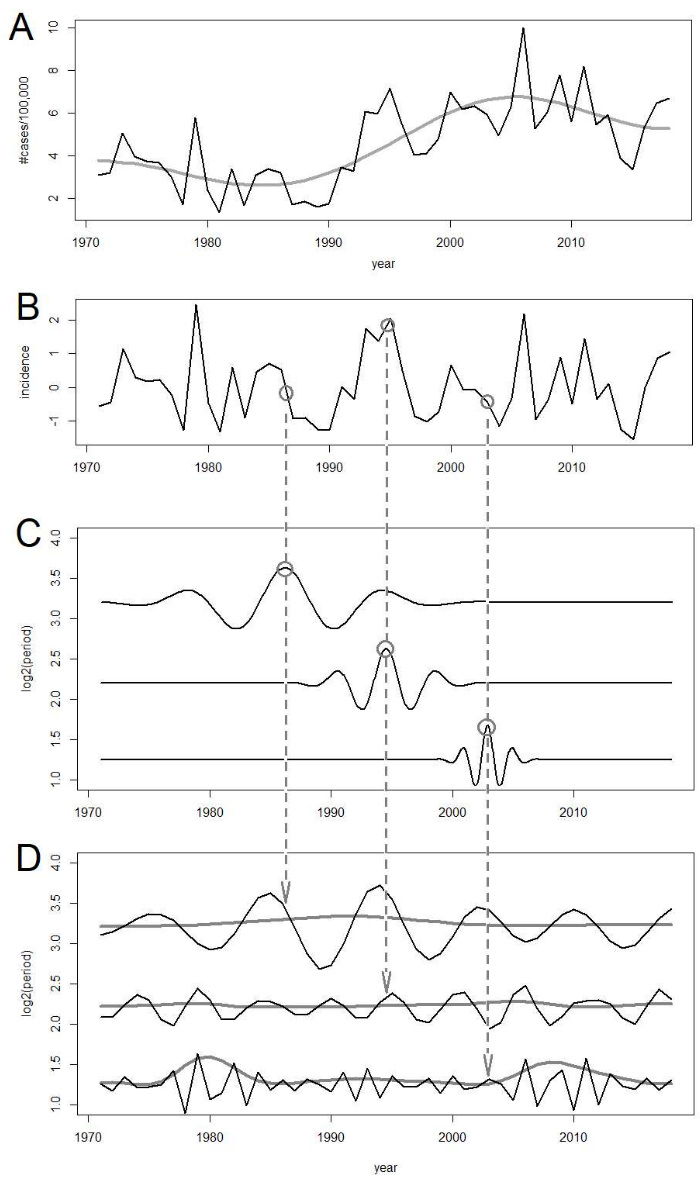

The analysis workflow.

This material illustrates the analysis in a necessarily simplified manner, and for all calculations with complex numbers only the real parts are shown. An interested reader can find a more erudite explanation of wavelet analysis elsewhere (e.g. in Torrense and Compo (1998) or Cazelles et al. (2008), and references therein).

The analysis workflow can be divided into the three steps: (1) data pre-processing, (2) wavelet transformation and (3) spectra comparison.

(1) An example of TBE incidence series is shown in (A). Fluctuations in the incidence could equally likely reflect variations in zoonotic risk as well as in an at-risk population background. The former can be attributed to cyclic or recurring processes in the zoonotic system, whereas the latter to changes in human population density, disease reporting, vaccination etc. that are, as a rule, non-periodic in nature. To neutralize the latter effects (which are largely region/country-specific) while retaining the desired periodicities, the data was (optionally) 'stationarized', i.e. modified to ensure that the mean and variance do not change over time, as shown in (B).

(2) It can readily be seen in (B) that both amplitude and frequency varied over time – for example, periods of high frequency 'spikes' (1978-84 and 2005-14) alternate with rather low-frequency periods. Our goal is to disentangle these oscillations in order to determine which frequency was present, and when and how strong it was over time. An elegant solution is wavelet transformation (WT) that enables decomposing of a time series (TS) into time/frequency space simultaneously. WT is defined as a convolution (an operation related to cross-correlation) of the TS with a special 'wavelet' function. The most commonly used Morlet wavelet is illustrated in (C) - it is a combination of a Sine wave and a Gaussian wave. How does it work? Assuming that the width of this wavelet is 10 yrs, one can find the correlation between this curve and a 10-yr segment of the analysed TS – i.e. to measure how much [amplitude] the TS at a given point of time (*t*) resembles a Sine wave of this width [frequency]. By sliding this wavelet along the TS one can then construct a new time series of the projection amplitude versus time (D). Next, one can let vary the 'scale' (*s*) of the wavelet by changing its width, and proceed bandwidth by bandwidth across the spectrum of oscillations as indicated in (C) and (D). The output of WT (W(*s*,*t*)) can be further analysed. Because the wavelet function is in general complex, W(*s*,*t*) is also complex and can then be divided into the real part and imaginary part, or amplitude and phase. Finally, one can define the wavelet power spectrum as the absolute value squared W(*s*,*t*). The grey lines in (D) indicate how the projected waves translate to power.

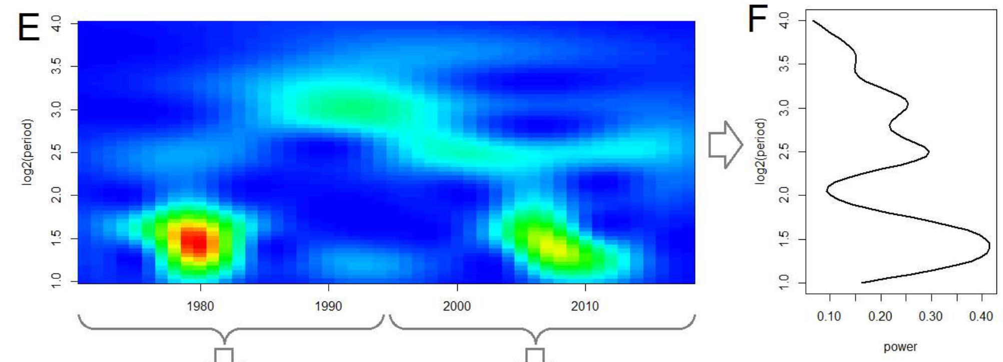

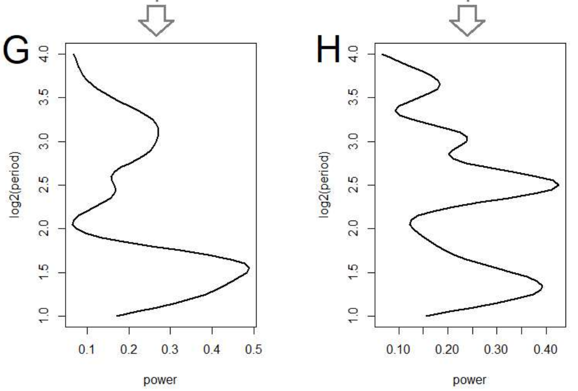

(3) A completed 'map' of the wavelet power spectrum is shown in (E). It illustrates how the spectrum of oscillations evolved over time. For example, the yellow-red areas indicate that high triennial oscillation activity occurred during 1978-84 and 2005-14, and that elevated decadal oscillations occurred during 1975-95. For comparison between geographical areas, the spectra were averaged along the time dimension (F). To evaluate whether there were significant changes during the course of climate warming, the period of observation was split into two equal length segments for which the average power spectra were calculated separately and compared to each other (G and H).

References.

Cazelles, B.; Chavez, M.; Berteaux, D.; Menard, F.; Olav Vik, J.; Jenouvrier, S.; Stenseth, N.C. Wavelet analysis of ecological time series. Oecologia 2008, 156, 287–304.

Torrence, C.; Compo, G.P. A practical guide to wavelet analysis. Bull. Amer. Meteorol. Soc. 1998, 79, 61–78.
